# Greenwashing and financial performance in public health firms: the mechanism of organizational legitimacy erosion

**DOI:** 10.3389/fpubh.2025.1565703

**Published:** 2025-03-25

**Authors:** Yaru Liang, Xiang Gao

**Affiliations:** ^1^School of Business, Shanghai Normal University Tianhua College, Shanghai, China; ^2^Research Center of Finance, Shanghai Business School, Shanghai, China

**Keywords:** greenwashing, corporate financial performance, organizational legitimacy, public health services, Chinese firms

## Abstract

**Introduction:**

Amid China’s pursuit of its “dual carbon” objectives and the escalating emphasis on Environmental, Social, and Governance (ESG) disclosure, corporate environmental responsibility has emerged as a critical regulatory and market concern. However, mounting institutional and stakeholder pressures have incentivized some firms to engage in greenwashing—strategically overstating or misrepresenting their environmental commitments—to sustain legitimacy and competitive positioning.

**Methods:**

This study empirically investigates the impact of greenwashing on corporate financial performance using panel data from 157 publicly listed Chinese public health firms between 2020 and 2022. A mediation model is utilized to identify the mechanism role of organizational legitimacy in affecting the relationship between greenwashing and corporate financial performance.

**Results:**

The findings reveal that greenwashing significantly undermines organizational legitimacy, which, in turn, leads to negative financial repercussions. Firms that overstate their environmental commitments experience diminished stakeholder trust, regulatory scrutiny, and reputational damage, ultimately eroding their financial performance.

**Discussion:**

By analyzing the indirect pathways through which greenwashing influences firm performance, this research advances theoretical discourse on corporate environmental accountability and legitimacy-based performance dynamics. Furthermore, the study offers actionable insights for public health enterprises, emphasizing the necessity of transparent and verifiable environmental communication to mitigate reputational risks and ensure sustainable financial outcomes. Additionally, it provides empirical evidence to inform policymakers in refining regulatory frameworks that enhance transparency in environmental reporting and foster substantive sustainability practices.

## 1 Introduction

In recent years, China has reinforced its commitment to sustainability through ambitious environmental initiatives, including its “dual carbon” goals, which aim to achieve peak carbon emissions by 2030 and carbon neutrality by 2060. At the same time, the government has tightened ESG disclosure regulations, requiring companies to enhance transparency regarding their environmental impact. While these policies are intended to drive genuine corporate sustainability, they may also create unintended pressures, leading some firms to engage in greenwashing (1). To meet regulatory expectations while keeping compliance costs low, certain companies may overstate or misrepresent their environmental efforts (2). This raises concerns about the credibility of corporate sustainability claims and their long-term effects on stakeholder trust and business performance.

Greenwashing is a form of corporate disinformation in environmental communication, where an organization disseminates exaggerated, misleading, or unsubstantiated claims regarding the environmental sustainability of its products, services, or overall operations. This practice aims to create a false perception of ecological responsibility, thereby obfuscating actual environmentally detrimental behaviors and practices. Greenwashing often serves to manipulate stakeholders and consumers by projecting a facade of corporate environmental responsibility without substantive actions to support such claims (3). From the perspective of stakeholder theory, while some researchers suggest that greenwashing might yield short-term benefits—such as an enhanced brand image, heightened consumer trust, and increased sales (4)—it can have detrimental long-term effects. Once exposed, greenwashing practices can severely damage a company’s reputation and erode consumer trust, ultimately producing adverse effects on firm performance (5, 6).

Recent studies have identified key mechanisms through which greenwashing detrimentally affects firms. First, the direct financial consequences of greenwashing can be significant. Firms may incur substantial costs due to legal disputes, regulatory fines, and the expenses of managing the resulting crisis, such as conducting public relations campaigns to repair their image. These financial burdens not only drain resources that could otherwise support core business activities but also harm the company’s reputation, further exacerbating the long-term financial damage (7). Second, greenwashing can significantly undermine investor confidence, as investors may perceive the firm’s environmental claims as deceptive or misleading. This loss of trust can lead to increased stock price volatility and may prompt investors to divest, further destabilizing the company’s financial position (8). Third, greenwashing has profound implications for consumer behavior. When consumers perceive a company’s environmental claims as deceptive or disingenuous, the result is a significant decline in both brand loyalty and trust (6). This erosion of trust extends beyond environmental concerns and spills over into broader perceptions of corporate integrity and product quality. This loss of trust has severe long-term implications, as it compromises consumer retention, a key driver of sustained business success. Notably, younger consumers, who are increasingly discerning and skeptical of environmental claims, are particularly vulnerable to abandoning brands that engage in greenwashing (9). The rise of social media has further amplified this scrutiny, making it increasingly difficult for firms to project an authentic “green” image without genuine commitment to sustainable practices.

However, relatively few studies have examined the transmission mechanisms through which greenwashing affects firm performance. A strand of research on the financial consequences of greenwashing has predominantly explored the contingent effects of local environmental regulations and media scrutiny in shaping this relationship (10). Such work suggests that greenwashing can have varying effects on a company’s financial performance depending on the strength of local environmental regulations and the extent of media coverage. Local regulations can either mitigate or exacerbate the negative financial effects of greenwashing, while media coverage increases public and investor scrutiny, further influencing the company’s financial outcomes. Chen and Dagestani (11) examined the relationship between greenwashing behavior and firm value, highlighting the role of board characteristics as a mediating variable. They found that the composition and governance structure of the board play a significant mediating role in the relationship between greenwashing and firm value. Effective board governance can influence how a company manages greenwashing risks and its overall value, with board characteristics affecting both the company’s environmental performance and its value.

While these studies provide valuable insights into the external factors moderating greenwashing’s financial impact and the internal governance mechanisms that mediate this relationship, they fall short in explaining the fundamental transmission channels through which greenwashing translates into financial consequences. Although prior research has acknowledged legitimacy as an important determinant of firm success, few studies have explicitly examined how greenwashing erodes legitimacy and, in turn, deteriorates financial performance. The present study innovatively considers the role of organizational legitimacy as a functionary channel in enabling the adverse effect of greenwashing on corporate financial performance. Using empirical methods, we attempt to confirm this proposal with data sourced from 137 listed Chinese public health related companies for the period 2020–2022. We refer to the tenets of legitimacy theory and signaling theory to support our model. According to legitimacy theory, to maintain legitimacy, organizations must align their actions and disclosures with societal norms and expectations (12). Legitimacy is a social construct reflecting the perceived alignment between organizational behavior and societal values. Significant deviations can threaten legitimacy, leading to increased scrutiny, heightened criticism, and potential sanctions. When a company engages in greenwashing, it constructs a deceptive narrative of environmental responsibility that may not align with its actual practices (3). This inconsistency between stated commitments and real actions heightens stakeholder skepticism, undermining perceptions of corporate integrity and ethical accountability (13). As legitimacy is fundamentally contingent on stakeholder trust and perceived alignment with societal expectations, such discrepancies can erode organizational credibility, exposing firms to reputational risks, regulatory scrutiny, and diminished stakeholder support. Based on signaling theory, Bjornali et al. (14) propose that a firm’s legitimacy serves as a crucial signal, conveying essential information to stakeholders. Legitimacy can yield financial advantages for firms. Such benefits include improved employee attraction and retention, enhanced growth opportunities, and reduced opposition from social activists and NGOs, as well as a minimized risk of regulatory fines, penalties, and sanctions (15).

This study offers several key contributions to the existing literature. First, based on stakeholder theory, we investigate the relationship between greenwashing and financial performance in Chinese public health firms. While previous research has mainly focused on developed countries, studies of this relationship in developing countries are relatively scarce. Thus, our investigation of greenwashing behavior in Chinese public health related firms offers valuable insights that can guide the regulation of such practices in other developing countries and further enrich stakeholder theory. Second, existing studies have not thoroughly examined the effect of greenwashing on firms’ financial performance, with a particular gap regarding the mechanism role of organizational legitimacy. We aim to bridge that gap by exploring how organizational legitimacy acts as a transmission mechanism between greenwashing and firms’ financial performance. In this way, we validate the applicability of legitimacy theory and signaling theory in the context of greenwashing.

## 2 Literature review and hypothesis development

### 2.1 Theoretical framework

In explaining the relationship between greenwashing and a firm’s financial performance, stakeholder theory and signaling theory provide key insights. Stakeholder theory posits that a firm’s financial success is contingent on maintaining trust and legitimacy with key stakeholders, including customers, investors, and regulators (16). Greenwashing disrupts this trust by creating a misalignment between a firm’s environmental claims and its actual practices, ultimately provoking skepticism and negative market reactions (8, 17). When stakeholders perceive inconsistencies in a firm’s sustainability commitments, they may withdraw support, leading to financial penalties such as declining investor confidence and consumer disengagement.

Signaling theory further explains this dynamic by emphasizing how firms communicate credibility and transparency through observable signals (14). Effective environmental responsibility serves as a positive signal to stakeholders, whereas greenwashing constitutes a deceptive signal that, when exposed, damages the firm’s broader corporate reputation. Such misleading signals heighten scrutiny, increase perceived investment risks, and contribute to deteriorating financial outcomes (8).

At the core of this relationship is organizational legitimacy, which serves as a mediating mechanism linking greenwashing to financial performance. Legitimacy theory asserts that organizations must align their behaviors with societal norms and values to secure long-term stakeholder support and operational stability (12). Greenwashing erodes legitimacy by fostering a discrepancy between corporate narratives and actual conduct, leading to reputational damage and regulatory pressures. From a resource-based view, corporate reputation is an intangible yet strategically valuable asset that strengthens competitive advantage (4). When a firm engages in greenwashing, it not only jeopardizes its legitimacy but also diminishes its reputational capital, triggering resource misallocation as firms attempt to repair their public image (18). This diversion of resources constrains the firm’s ability to pursue sustainable growth, exacerbating the negative financial consequences of greenwashing (19).

By integrating stakeholder theory, signaling theory, legitimacy theory, and the resource-based view, this study provides a comprehensive framework for understanding the mechanisms through which greenwashing affects firm performance. These interconnected processes—erosion of stakeholder trust, reputational damage, and resource inefficiencies—collectively amplify the financial risks associated with greenwashing, underscoring the importance of authentic environmental responsibility for corporate sustainability.

### 2.2 Greenwashing and corporate financial performance

A company’s financial performance largely depends on its trust relationship with external stakeholders, such as customers, investors, suppliers, and the public. According to stakeholder theory, a company must balance the expectations and needs of various stakeholders while pursuing its financial goals (16). However, when a company engages in greenwashing, it intentionally undermines this trust relationship (20). This can trigger negative market reactions and trust crises, leading to consumer boycotts and investor sell-offs, which affect the company’s revenues and stock performance (8).

Additionally, according to signaling theory, every public action a company takes sends a message to the market about its strategic intentions and internal conditions. Greenwashing acts as a false signal, and once exposed, it not only leads to skepticism about the company’s environmental commitments but also raises broader doubts about the company’s overall transparency and integrity (14). Research has shown that such behavior can lead to a loss of credibility and trust, as investors and stakeholders might perceive the company as less reliable and more deceptive (17). This lack of integrity can be interpreted as a sign of poor governance and decision-making, which might influence investment decisions and future cashflow expectations (8). The resulting uncertainty also increases the company’s capital costs and stock volatility, further deteriorating its financial performance (7). From the theories above, we can say that greenwashing hurts the trust between stakeholders, sends bad signals, raises capital costs, lowers shareholder value, and ultimately causes the company’s finances to get worse.

*H1*: Greenwashing decreases firms’ financial performance.

### 2.3 The mechanism of organizational legitimacy erosion

According to legitimacy theory, organizations must align their actions, strategies, and disclosures with societal norms and expectations to maintain credibility (12). Legitimacy is a socially constructed perception of consistency between corporate behavior and broader value systems. When discrepancies arise between a company’s claims and actual practices, stakeholders may view this as deceptive, leading to diminished trust and reputational damage (21, 22).

From a stakeholder theory perspective, a decline in legitimacy can provoke public scrutiny, regulatory pressure, and potential sanctions, all of which heighten operational risks. Agency theory further suggests that firms facing such crises often incur higher compliance and reputational management costs to restore confidence (23–25). Additionally, the resource-based view underscores reputation as a vital intangible asset that sustains competitive advantage (4). Erosion of corporate credibility can weaken consumer loyalty, reduce market share, and negatively impact financial performance (1, 26).

Building on these theoretical foundations, we argue that legitimacy mediates the relationship between greenwashing and financial outcomes. Firms engaging in misleading sustainability claims risk losing stakeholder confidence, facing stricter regulatory oversight, and weakening their competitive standing—all of which carry tangible financial repercussions.

*H2*: Organizational legitimacy mediates greenwashing’s effect on firms’ financial performance.

## 3 Research design

### 3.1 Sample selection

The purpose of this study is to investigate how greenwashing affects firms’ financial performance through legitimacy. The data come from listed Chinese public health related companies for the years 2020–2022. COVID-19 notably affected business operations and environmental practices in China from 2020 to 2022, a period commonly referred to as the pandemic years in China. We focus on the period 2020–2022 to ensure that the sample companies were operating in a consistent macroeconomic environment. This helps minimize the confounding effects of external factors on the results, ensuring the validity and reliability of the findings.

Furthermore, we select balanced panel data for the sample to ensure the robustness and reliability of the results. Data collection for this study involves the iFinD, Bloomberg, and Asset4 databases. Due to varying data availability across different databases, not all companies have complete information from all sources. Ultimately, we select a sample of 157 listed A-share Chinese public health-related companies.

We use W.A. Edwards’ sample size calculation formula as defined in Equation 1 to confirm the rationality of the selected sample size:


(1) $$N=\frac{Z^{2}\times P\times \left(1-P\right)}{E^{2}},$$

where *N* is the required sample size, and *Z* is the *Z*-score corresponding to the chosen confidence level. We typically determine *P*, the proportion to estimate, based on prior experience or expectations. *E* is the desired margin of error. Validation reveals that the selected sample size for this study is reasonable.

The data processing steps are as follows: To avoid sector specific biases, we first exclude companies in the financial sector. We also remove firms labeled ST, *ST, and PT to concentrate on stable companies. We also excluded data from companies with missing or anomalous values to uphold the quality and reliability of the dataset.

The data for this analysis were sourced from three main databases: corporate financial data and legitimacy were obtained from the iFinD database, and greenwashing-related data were collected from Bloomberg’s ESG and Asset4 ESG databases.

### 3.2 Dependent variables

Accounting-based measurements such as return on assets (ROA) and return on equity (ROE) are widely regarded as effective indicators of a company’s financial performance, reflecting its profitability and efficiency in resource utilization (27, 28). ROA, which measures a company’s ability to generate profits from its total assets, provides a comprehensive view of how efficiently a company is utilizing its resources to produce earnings (29). Therefore, ROA is suitable for evaluating a company’s overall financial performance (30). Furthermore, ROE, which indicates the return generated on shareholders’ equity, is often used to assess a company’s financial stability and profitability from the perspective of equity investors (31). In this study, we use ROA as the main measure of a company’s financial performance while ROE is used for stability testing, providing a robust framework for analyzing both operational efficiency and shareholder value creation. However, ROA and ROE, as accounting-based metrics, are inherently short-term oriented, making them susceptible to earnings volatility and limiting their ability to fully reflect a firm’s long-term financial sustainability and strategic performance.

### 3.3 Independent variable

Greenwashing is conceptualized as the discrepancy between a firm’s ESG disclosure score and its actual ESG performance (13). Following Yu et al. (32), we use Bloomberg ESG score ($ESG_{dis,i,t}$) as the nominal disclosure score and the Asset4 ESG score ($ESG_{real,i,t}$) as the actual performance score. The greenwashing score (GW) for firm i at year t is calculated by the following Equation 2:


(2) $$\begin{array}{l} Greenwashing_{i,t}=\left(\frac{ESG_{dis,i,t}-\bar{ESG_{dis}}}{\sigma_{dis}}\right)-\\ \;\left(\frac{ESG_{real,i,t}-\bar{ESG_{real}}}{\sigma_{real}}\right), \end{array}$$

where $\bar{ESG_{dis}}$ and $\bar{ESG_{real}}$ are the mean value for disclosed and real ESG score, and $\sigma_{dis}$ and $\sigma_{real}$ are the corresponding standard deviations of the two scores.

The ESG disclosure-minus-performance approach is a widely recognized measure but comes with certain limitations. Divergences in ESG rating methodologies, as seen in Bloomberg and Asset4, can lead to inconsistencies, while self-reported data may introduce bias in assessment. Additionally, ESG scores are influenced by both external forces, such as market fluctuations, and firm-specific elements, causing variations across firms and over time. Since ESG performance improvements materialize over extended periods, whereas disclosures can be modified more promptly due to corporate decisions or external pressures, this discrepancy may lead to potential distortions. Applying firm and year fixed effects can enhance measurement precision by accounting for hidden firm-level reporting patterns and temporal variations.

### 3.4 Mechanism variable

The measurement of organizational legitimacy has been widely examined in the literature, with three predominant approaches emerging: code adoption, organizational affiliation, and media perception (33). Code adoption reflects a firm’s compliance with institutional norms and regulatory frameworks; however, it primarily captures formal adherence rather than stakeholders’ subjective perceptions of legitimacy. Organizational affiliations, including external accreditations and professional memberships, serve as quality signals but may not fully encapsulate the broader societal acceptance of a firm. In contrast, media perception offers a more dynamic and externally validated measure of legitimacy, as media narratives influence public opinion, shape stakeholder trust, and serve as a conduit for societal judgment (34). Given that media coverage both reflects and constructs public legitimacy assessments, it provides a more comprehensive and real-time proxy for legitimacy. Prior studies have established the validity of media sentiment as a measure of organizational legitimacy by demonstrating its alignment with stakeholder perceptions and its predictive power in legitimacy-related outcomes (35). Therefore, building upon this established theoretical and empirical foundation, we adopt media sentiment analysis as a legitimacy proxy, acknowledging its robustness in capturing evolving legitimacy dynamics and its broader relevance in institutional and stakeholder discourse.

We measure organizational legitimacy (LEG) using sentiment data from the iFind database, analyzing media coverage of each firm. Specifically, we compute the Janis-Fadner coefficient (36) based on the distribution of positive, negative, and neutral news articles (See Equation 3 for the formula for computation). This index ranges from 1 (indicating exclusively favorable reports) to −1 (reflecting entirely critical coverage), with legitimacy increasing as positive articles dominate and decreasing with negative coverage. To ensure accuracy, firms with incomplete sentiment data are excluded from the sample.


(3) $$Janis–Fadner\;Coefficient=\{\begin{array}{c} \frac{e^{2}-ec}{t^{2}},e>c\\ \frac{ec-ec^{2}}{t^{2}},e<c\\ 0,e=c \end{array},$$

where *e* means positive news, *c* means negative news, and *t* is the sum of positive and negative news.

### 3.5 Control variables

In addition to the abovementioned core variables, we incorporate control variables based on the literature. There are many studies of the effects of factors such as firm size, leverage, and firm growth on the relationship between greenwashing and firms’ financial performance.

Firm size influences a company’s ability to seize capital-intensive opportunities, as greater resources provide a competitive edge. This advantage not only supports economies of scale but also enhances market positioning in imperfect competition (37). Empirical evidence further validates this relationship (38). To control for firm size (SIZE), we employ the natural logarithm of total assets as a proxy.

Leverage, represented by the debt ratio (total debt to total assets), reflects a firm’s financial structure and risk exposure (39). While greater leverage increases financial risk, it can also enhance returns by leveraging tax advantages and other financial incentives. To account for this effect, we incorporate the debt ratio (LEV) as a control variable in our analysis.

Firm growth, often gauged by the growth rate of operating income, indicates a firm’s ability to expand and generate higher revenues over time. This growth rate is an indicator of a company’s ability to reinvest in its core business areas, innovate, and maintain a competitive advantage (40). We incorporate the growth rate of operating income in our model to control for the effect of firm growth (GROWTH) on firms’ financial performance.

Institutional investors play a vital role in corporate governance by enhancing oversight and providing strategic expertise, which can positively impact firm performance (41). To reflect this influence, we control for institutional ownership (INST) using the percentage of shares held by these investors.

The number of employees (EMP), as a reflection of a firm’s human capital and operational scale, is another vital determinant of financial performance. Larger workforces can indicate a greater capacity to execute complex projects and maintain operational stability (42). We use the natural logarithm of the number of employees to include the effect of human capital.

To ensure a thorough analysis of the link between greenwashing and financial performance, we incorporate these control variables. Additionally, firm and time fixed effects are included to account for unobserved heterogeneity.

### 3.6 Empirical specification

The central premise of this model is that greenwashing impacts firm financial performance indirectly by shaping organizational legitimacy. In Baron and Kenny’s (43) classical model, a variable functions as a mediacor when it meets the following three conditions.

Condition (a): the independent variable should significantly influence organizational legitimacy, the mediating variable. This indicates that the independent variable can indirectly affect the dependent variable through the mediator. If there is no significant relationship between the independent variable and the mediating variable, the potential mediation effect cannot be established. Condition (b): The independent variable must significantly affect the mechanism variable of organizational legitimacy. This ensures that the independent variable influences the mechanism variable, which in turn affects the dependent variable. Without a significant relationship between the independent variable and the mechanism variable, the potential mediation effect cannot occur. Condition (c): when both the independent variable and the mediating variable are included in the regression model, a significant effect of the mediating variable on the dependent variable suggests the presence of a mediation effect. If the independent variable remains significant, it indicates partial mediation; if the independent variable is no longer significant, it suggests full mediation.

To test these conditions, researchers typically use three regression models. First, we employ Equation 4 to test the validity of condition (a) by examining whether the independent variable significantly affects the dependent variable. Specifically, the coefficient a1 captures the effect of the independent variable (GW*_i,t_*) on the dependent variable (FP*_i,t_*). If $a_{i}$ is statistically significant, it establishes a direct relationship between the independent and dependent variables.


(4) $$FP_{i,t}=a_{0}+a_{1}GW_{i,t}+\sum_{i=1}^{5}a_{i}control+\varepsilon_{i,t}.$$

Second, we utilize Equation 5 to test condition (b) by assessing whether the independent variable significantly affects the mechanism variable. The coefficient $\beta_{1}$ represents the influence of GW*_i,t_* on LEG*_i,t_*. If the estimate of $\beta_{1}$ is significant, it demonstrates that the independent variable has a meaningful effect on the mechanism variable, fulfilling the requirement for mediation.


(5) $$LEG_{i,t}=\beta_{0}+\beta_{1}GW_{i,t}+\sum_{i=1}^{5}\beta_{i}control+\sigma_{i,t}.$$

The third test is performed by Equation 6 for validating condition (c), where we check whether the relationship between the mechanism variable and the dependent variable remains significant after including the independent variable. In this case, the coefficient $\gamma_{2}$ quantifies the effect of the mechanism variable (LEG*_i,t_*) on the dependent variable (FP*_i,t_*), while $\gamma_{1}$ represents the effect of the independent variable (GW*_i,t_*). If $\gamma_{2}$ is significant and $\gamma_{1}$ remains significant, partial mediation is indicated. However, if $\gamma_{2}$ is significant and $\gamma_{1}$ is insignificant, full mediation is confirmed. These three conditions are tested sequentially in regression:


(6) $$FP_{i,t}=\gamma_{0}+\gamma_{1}GW_{i,t}+\gamma_{2}Legitmancy_{i,t}+\sum_{i=1}^{5}\gamma_{i}control+\theta_{i,t}.$$

## 4 Regression results and discussion

### 4.1 Summary statistics

Table 1 presents the descriptive statistics for the variables. The average ROA is 0.062, with a standard deviation of 0.077, and the data range from −0.266 to 0.541. The average value for greenwashing is 0.000, suggesting that Chinese public health related companies generally do not exhibit greenwashing behavior. However, the standard deviation of this indicator is 0.780, and the data range from −2.421 to 2.917, indicating that there might be significant differences among companies. The average level of the legitimacy index is 0.209, with a standard deviation of 0.617, indicating noticeable differences in legitimacy among Chinese public health related firms as well. For control variables, the average growth rate of firm growth is 0.153 (the range is from −0.859 to 4.478), with a standard deviation of 0.434. This indicates that some Chinese public health companies demonstrate significant growth potential, while others might face negative growth challenges, reflecting the intense competition among Chinese public health related enterprises. Firm size and employee size range from 4.605 to 8.424 and from 2.646 to 5.586, respectively, indicating a relative concentration of Chinese public health related enterprises in terms of both employee number and company size. The average leverage ratio of 0.473, with a standard deviation of 0.187, indicates significant variations in debt utilization among enterprises. Institutional ownership has an average of 53.7%, with a standard deviation of 0.198, indicating that institutional ownership is quite common among Chinese public health related enterprises.

**Table 1:** Descriptive statistics.

Variable	*N*	Mean	SD	Min	Max
ROA	471	0.062	0.077	−0.266	0.541
GW	471	0.000	0.780	−2.421	2.917
LEG	471	0.209	0.617	−1.000	1.000
GROWTH	471	0.153	0.434	−0.859	4.478
SIZE	471	6.436	0.682	4.605	8.424
EMP	471	4.067	0.572	2.646	5.586
LEV	471	0.473	0.187	0.014	0.898
INST	471	0.537	0.198	0.042	0.965

### 4.2 Correlation analysis

Table 2 reports Pearson’s correlation coefficients, providing valuable insights into the interrelationships among the key variables. Using Cohen’s (44) classification, correlations between 0.4 and 0.6 indicate moderate associations, 0.2 to 0.4 represent weak associations, and 0 to 0.2 signify very weak associations. The results in this study predominantly fall within the very weak to weak ranges, reflecting nuanced dynamics among greenwashing, legitimacy, and profitability.

**Table 2:** Correlation coefficients.

	ROA	GW	LEG	GROWTH	SIZE	EMP	LEV
GW	−0.060						
LEG	0.097	−0.053					
GROWTH	0.290	0.015	0.075				
SIZE	−0.099	−0.022	0.026	−0.088			
EMP	−0.082	−0.026	0.087	−0.014	0.541		
LEV	−0.420	−0.053	0.048	0.000	0.453	0.473	
INST	0.113	0.022	0.055	−0.049	0.285	0.307	0.198

For instance, the correlation coefficient of −0.06 between ROA (return on assets) and GW (greenwashing) indicates a very weak inverse relationship, suggesting that as greenwashing slightly increases, profitability may marginally decrease. Even though this link is not very strong, it fits with signaling theory (45). Greenwashing, as a form of misleading sustainability communication, sends negative signals to consumers and investors, potentially damaging trust and stakeholder loyalty. Furthermore, from a resource-based view perspective, greenwashing can dilute the firm’s reputational capital, which is a key intangible resource that drives competitive advantage and long-term profitability (4).

Similarly, the correlation coefficient of −0.053 between GW (greenwashing) and LEG (legitimacy) reveals a very weak negative association. This suggests that as greenwashing increases, a firm’s legitimacy with stakeholders tends to diminish. This finding supports the tenets of legitimacy theory. Perceived inconsistencies between a company’s stated sustainability goals and its actual practices can create a legitimacy gap, eroding trust and stakeholder support. From the lens of stakeholder theory, greenwashing risks alienating critical stakeholder groups, such as consumers, investors, and regulators, thereby compromising the firm’s ability to build long-term relationships and secure strategic resources (4).

The weak correlation of 0.097 between LEG (legitimacy) and ROA (return on assets) suggests a positive association between higher legitimacy and improved profitability. According to the resource dependence theory (46), firms with more legitimacy often get more reputational capital, more trust from stakeholders, and easier access to key resources. Such legitimacy enables firms to navigate regulatory environments more effectively, reduce transaction costs, and foster favorable relationships with key stakeholders. From the perspective of social capital theory, legitimacy can also enhance a firm’s network position, enabling it to access critical information, funding, and opportunities that contribute to financial performance (47).

We conducted multicollinearity diagnostics to ensure the validity of these results. The mean variance inflation factor (VIF) values, with the highest being 1.25, fall well below the commonly accepted threshold of 10 (48). This confirms that there exits no major multicollinearity problems. This supports the reliability of the dataset and the decision to include all variables in the regression model. The low VIF values further suggest that collinearity does not confound the observed relationships, leading to a more reliable interpretation of the regression outcomes.

### 4.3 Regression results and the role of organizational legitimacy

Table 3 presents the regression results. There is a statistically significant negative effect on ROA at the 5% level, as shown in Column (1), with a coefficient of −0.009. This supports H1: greenwashing hurts companies’ bottom lines. Following the mechanism analysis framework, Condition (a) is satisfied, as greenwashing significantly affects ROA. With a coefficient of −0.119 and a 5% level of significance, the estimates in column (2) show that greenwashing has a big effect on legitimacy. This means that condition (b) is also met. This indicates that greenwashing impacts the underlying influence channel of legitimacy. Lastly, in column (3), when both the mechanism variable of organizational legitimacy and the independent variable of greenwashing are added to the model, legitimacy has a significant positive relationship with ROA at the 1% significance level. Greenwashing, on the other hand, still has a significant negative relationship with ROA. This suggests partial mediation. These results confirm H2, which states that organizational legitimacy mediates the effect of greenwashing on firms’ financial performance.

**Table 3:** Baseline regression results.

	(1)	(2)	(3)
	ROA	LEG	ROA
GW	−0.009** (−2.112)	−0.119** (−2.094)	−0.012** (−2.273)
LEG			0.008* (1.721)
GROWTH	0.042*** (7.321)	0.024 (0.305)	0.001*** (6.944)
SIZE	0.002 (0.274)	−0.030 (−0.383)	0.007 (0.943)
EMP	0.069* (1.723)	−0.679 (−1.249)	0.140*** (2.899)
LEV	−0.003*** (−6.377)	0.006 (0.913)	−0.003*** (−5.615)
INST	0.004 (0.018)	3.508 (1.205)	−0.014 (−0.401)
Constant	−0.100 (−1.066)	0.847 (0.668)	0.407 (0.196)
Firm fixed effect	YES	YES	YES
Year fixed effect	YES	YES	YES
*N*	471	471	471
Adj. *R*^2^	0.693	0.137	0.746

*p < 0.1, **p < 0.05, ***p < 0.01. Values in parentheses represent *t*-statistics.

### 4.4 Addressing the endogeneity issue

Our previous research findings show that greenwashing can negatively affect corporate financial performance. Even so, we are aware that there are some possible biases, like selection bias and reverse causality, that could introduce endogeneity and change the results of the regression analysis. To lessen the impact of these outside factors, we use strong techniques to check and confirm our regression analysis. These include the instrumental variables approach and the replacement of the dependent variable. Table 4 summarizes the detailed results.

**Table 4:** Endogeneity and robustness test results.

Variable	(1) 1st stage GW	(2) 2nd stage ROA	(3) ROE	(4) Legitimacy	(5) ROE
GW		−0.074* (−1.870)	−0.025*** (−2.864)	−0.119** (−2.094)	−0.036*** (−3.235)
IV (L.GW)	0.133* (1.780)				
LEG					0.020* (1.926)
Constant	−1.366 (−0.790)	−0.051 (−0.380)	−0.955*** (−4.932)	0.847 (0.668)	4.967 (1.099)
Controls	YES	YES	YES	YES	YES
Firm fixed effect	YES	YES	YES	YES	YES
Year fixed effect	YES	YES	YES	YES	YES
*N*	314	314	417	417	417
*R* ^2^	0.823	0.841	0.783	0.437	0.803

*p < 0.1, **p < 0.05, ***p < 0.01. Values in parentheses represent *t*-statistics.

Building on Chen and Dagestani (11), we apply the lagged first-order greenwashing as an instrumental variable in the 2SLS regression. Table 4 reports the estimation results. In the first stage (column 1), the lagged greenwashing variable shows a strong association, with a coefficient of 0.133 at a statistically significant level. The second stage (column 2) reveals a negative and significant impact of greenwashing, with a coefficient of −0.074. These findings remain robust, confirming that our approach effectively mitigates potential endogeneity.

Next, we assess the robustness of the regression results by substituting the dependent variable. We specifically replace ROA with ROE. In column (3), there is a negative relationship between ROE and greenwashing at the 10% significance level. Column (4) further supports this finding with a significant negative coefficient (−0.119) for greenwashing. After considering the coefficients in columns (3) and (4), column (5) shows a strong positive correlation between legitimacy and ROE at the 1% significance level. On the other hand, column (5) also shows a strong negative correlation between greenwashing and ROE. Various robustness tests substantiate our regression results, demonstrating their plausibility.

### 4.5 Firm heterogeneity analysis

Environmental scrutiny varies based on a company’s pollution levels, allowing us to classify firms into heavily polluting (HPC) and non-polluting (non-HPC) categories. Companies with significant environmental impact encounter stronger demands to maintain legitimacy, which drives their greenwashing behaviors (49). Based on the experimental data in column (1) of Table 5, it is evident that the impact of greenwashing (GW) on return on assets (ROA) is greater for heavily polluting firms compared to low-polluting firms. One possible explanation for this result is that heavily polluting firms are subject to greater scrutiny from stakeholders, such as regulators, investors, and consumers, who closely monitor their environmental practices. This heightened attention amplifies the financial risks and repercussions associated with greenwashing. Conversely, low-polluting firms face comparatively lower scrutiny, reducing the financial impact of their greenwashing behavior (11). The study uses the Chow test to confirm this variation further. The *p*-value of 0.874 for the coefficient difference between the heavily polluting (HPC) and non-polluting (non-HPC) groups shows that the difference is not statistically significant.

**Table 5:** Results of firm heterogeneity analysis.

	(1)		(2)	
	HPC	Non-HPC	CB4	Non-CB4
	ROA	ROA	ROA	ROA
GW	−0.013 (−1.450)	−0.009* (−1.760)	−0.015** (−2.580)	−0.008 (−1.520)
Constant	−0.339 (−1.230)	−0.093 (−0.990)	−1.706** (−2.330)	−0.107 (−0.990)
Controls	Yes	Yes	Yes	Yes
Firm fixed effect	Yes	Yes	Yes	Yes
Year fixed effect	Yes	Yes	Yes	Yes
*N*	129	342	90	381
Adj. *R*^2^	0.722	0.687	0.819	0.682
*P* (Chow Test)	0.874		0.548	

*p < 0.1, **p < 0.05. Values in parentheses represent *t*-statistics.

In addition, we examined the regulatory influence of third-party reviewers, specifically the Big Four audit firms, by categorizing companies into two groups: those audited by the Big Four (CB4) and those not audited by them (non-CB4). In the context of the relatively weak regulations and supervision over auditing in China, the involvement of Big Four firms plays a crucial role in enhancing the credibility of corporate financial and social responsibility reports. This increased credibility helps to mitigate greenwashing behaviors among firms (50). Based on the experimental data shown in column (2) of Table 5, greenwashing has a negative effect on the financial performance of companies that are audited by the Big Four firms. This effect is significant at the 5% level. In contrast, for companies not audited by the Big Four, the effect of greenwashing on financial performance is non-significant, though still negative. Furthermore, the magnitude of the negative impact of greenwashing on financial performance is greater for firms audited by the Big Four than for those not audited by them. One possible explanation for this is that companies audited by the Big Four are subject to more rigorous scrutiny by their auditors, leading to more credible and reliable social responsibility reports being disclosed to stakeholders (52). The increased scrutiny heightens the probability of identifying any instances of greenwashing. Consequently, if stakeholders—such as investors or consumers—discover greenwashing, they are likely to experience greater disappointment with companies audited by the Big Four due to the higher expectations of transparency and accuracy. In contrast, companies not audited by the Big Four may face less stringent oversight, which results in lower levels of stakeholder disappointment when greenwashing is identified (51). Like before, the study again uses the Chow test to test the existence of this variation. The test statistic indicates a *p*-value of 0.548 for the coefficient difference between companies audited by the Big Four and those not audited by them (non-CB4), which means the difference is not statistically significant.

## 5 Conclusion

Selecting Chinese companies related to public health as the research sample, this study investigates the mechanism role of organizational legitimacy in the relationship between greenwashing and firms’ financial performance. Using a two-way fixed-effects model and a series of robustness tests, we find that greenwashing poses a threat to organizational legitimacy. In turn, legitimacy decreases financial performance.

This study’s contribution to the theoretical literature is twofold. On the one hand, we explore the connection between greenwashing and firms’ financial performance in Chinese public health companies based on stakeholder theory and signaling theory. While much of the existing literature has focused on developed nations, there is a lack of research examining this relationship in developing countries. By analyzing greenwashing behaviors in Chinese public health related firms, this study offers significant insights that could help shape greenwashing regulations in other emerging economies. On the other hand, existing research has not fully explored the effect of greenwashing on financial performance, especially in terms of the mechanism role of organizational legitimacy. We seek to fill this gap by investigating how organizational legitimacy serves as an intermediary between greenwashing and firms’ financial performance. In this way, we validate the applicability of legitimacy theory.

This study examines industry practices from three key perspectives, with a particular emphasis on China’s public health sector, which plays a pivotal role in maintaining national well-being. From the standpoint of corporate strategy and reputation management, our findings underscore the critical importance of authenticity in corporate social responsibility claims, particularly for public health firms. Given their essential function in safeguarding societal health, any erosion of organizational legitimacy due to greenwashing could severely weaken public trust, reduce confidence among patients and institutional stakeholders, and invite regulatory scrutiny, ultimately undermining financial performance. To mitigate these risks, firms in this sector should prioritize genuine and transparent sustainability initiatives, rather than relying on superficial environmental claims. Long-term legitimacy, built upon authenticity, not only protects corporate reputation but also enhances financial resilience, making it an indispensable strategic priority for firms aiming to sustain competitive advantage in an increasingly environmentally conscious market.

In terms of government oversight, this study highlights the urgent need for stricter regulatory enforcement against greenwashing in China’s public health industry. As these firms are deeply embedded in public health systems and policy frameworks, deceptive environmental claims can mislead consumers, distort market competition, and compromise national sustainability objectives. Policymakers should therefore implement more rigorous disclosure standards and verification mechanisms to ensure that public health firms substantiate their environmental commitments with verifiable and credible evidence. Strengthening transparency requirements and introducing third-party audits could serve as an effective deterrent against misleading sustainability claims while fostering a more responsible and accountable corporate sustainability landscape, in alignment with China’s broader green development strategy.

From an investment perspective, this study provides a comprehensive framework for assessing the financial risks associated with corporate greenwashing. In China’s public health sector, where corporate legitimacy is closely tied to regulatory approvals, public procurement, and long-term institutional partnerships, misleading green claims may serve as a precursor not only to financial underperformance but also to heightened regulatory and reputational risks. Consequently, investors should integrate an assessment of environmental authenticity into their risk evaluation models, ensuring that firms’ sustainability commitments are both credible and aligned with long-term financial stability.

Future studies can improve upon the acknowledged limitations. First, there is no universally accepted measurement framework for organizational legitimacy in the literature. Various studies employ different approaches and definitions, leading to inconsistencies in how legitimacy is quantified. This makes it difficult to directly compare results across studies. Future research could contribute to the field by establishing a standardized metric for legitimacy, which would allow for more accurate and comparable analyses across different contexts. Second, there is room for further exploration of other factors that could influence the relationship between greenwashing and firms’ financial performance. For instance, consumer behavior, regulatory pressure, or competition intensity in a particular market could influence the outcomes of green-washing practices. Future studies could examine these factors to provide a more nuanced understanding of how and when greenwashing affects financial outcomes. Third, this study does not focus on a specific industry; thus, we are unable to consider whether the relationship between greenwashing and financial performance varies across different sectors. There may be differences in how the market works, the rules that apply, and what customers expect from each industry. These factors may affect how organizational legitimacy affects this relationship. Future research could address this gap by conducting industry-specific studies to determine whether the mechanisms at play differ across sectors. By addressing the abovementioned limitations, future research can provide a more comprehensive and refined understanding of the complex dynamics of greenwashing and company performance.

## Data Availability

The data analyzed in this study is subject to the following licenses/restrictions: they can be accessed through paid databases. Requests to access these datasets should be directed to service@csmar.com.
